# Short-term inspiratory muscle training, aerobic exercise, and detraining in women with COPD: a randomized controlled trial

**DOI:** 10.1186/s13102-026-01964-7

**Published:** 2026-08-08

**Authors:** Yasemin Ari Yilmaz, Mehmet Ismail Tosun, Erkan Demirkan, Abdulsamet Efdal, Hilal Boyaci, Irem Eker Arici, Yeliz Ay Yildiz, Hazel Kara Gunaydin, Mustafa Arici, Milan Markovic, Tomasz Kowalski

**Affiliations:** 1 Department of Pulmonary Diseases, Faculty of Medicine, Hitit University, Çorum, Türkiye; 2 Department of Physical Education and Sports, Faculty of Sport Sciences, Hitit University, Çorum, Türkiye; 3 Department of Coaching Education, Faculty of Sport Sciences, Hitit University, Çorum, Türkiye; 4 Department of Environmental Protection Technologies, Health Services Vocational School, Hitit University, Çorum, Türkiye; 5 Department of Pulmonary Diseases, Hitit University Çorum Erol Olçok Training and Research Hospital, Çorum, Türkiye; 6 Department of Coaching Education, Faculty of Sport Sciences, Alanya Alaaddin Keykubat University, Antalya, Türkiye; 7 Department of Recreation, Faculty of Sport Sciences, Hitit University, Çorum, Türkiye; 8 Faculty of Sport and Physical Education, University of Priština in Kosovska Mitrovica, Leposavić, Serbia; 9 Department of Physiology, Institute of Sport-National Research Institute, Warsaw, Poland

**Keywords:** Chronic obstructive pulmonary disease, Inspiratory muscle training, Aerobic exercise, Detraining, Pulmonary rehabilitation

## Abstract

**Background:**

Inspiratory muscle dysfunction, exercise intolerance, dyspnea, and reduced well-being contribute to the burden of chronic obstructive pulmonary disease (COPD). We compared the short-term effects of inspiratory muscle training (IMT), aerobic exercise (AE), combined IMT and AE (IMT_AE_), placebo IMT, and usual care on respiratory, functional, and patient-reported outcomes in women with stable COPD and examined early changes during two weeks after training cessation.

**Methods:**

In this single-center, five-arm, partially blinded randomized controlled trial, 68 women with stable GOLD stage I–II COPD were randomized; 59 completed all assessments and were included in the per-protocol analysis. The four-week intervention was followed by assessments 7 and 14 days after cessation. The prespecified primary outcome was baseline-to-post change in maximal inspiratory pressure (MIP). Secondary outcomes were maximal expiratory pressure (MEP), peak inspiratory flow rate (PIFR), inspiratory volume (IV), forced vital capacity (FVC), forced expiratory volume in 1 s (FEV₁), FEV₁/FVC, 6-min walk test (6MWT) distance, post-exercise Borg dyspnea, and WHO-5 well-being. The primary analysis tested the baseline-to-post group × time interaction for MIP using a 5-group × 2-time mixed-design repeated-measures analysis of variance. Four-time analyses examined secondary and detraining trajectories; additional Welch change-score comparisons were exploratory and unadjusted for multiplicity.

**Results:**

The primary MIP interaction was significant (F(4, 54) = 28.969, *p* < 0.001, ηp² = 0.682). Secondary four-time interactions were significant for MIP, MEP, PIFR, IV, 6MWT distance, FVC, FEV₁, Borg dyspnea, and WHO-5, but not FEV₁/FVC. Combined training produced the largest improvements in inspiratory performance, walking distance, dyspnea, and well-being. Its 6MWT gain, however, was modest relative to commonly reported minimal clinically important differences. Several outcomes declined during detraining.

**Conclusions:**

Within this sample of women with stable GOLD stage I–II COPD, combined IMT and AE produced the broadest short-term response across respiratory, functional, and patient-reported outcomes. Larger, prospectively registered trials with longer intervention and follow-up periods are needed to confirm these findings.

**Trial registration:**

ClinicalTrials.gov, NCT07604961; first submitted on 17 May 2026 and first posted on 22 May 2026 (retrospectively registered).

**Supplementary Information:**

The online version contains supplementary material available at https://doi.org/10.1186/s13102-026-01964-7.

## Introduction

Chronic obstructive pulmonary disease (COPD) is a progressive respiratory disorder characterized by persistent airflow limitation, chronic respiratory symptoms, and reduced functional capacity [1]. Its clinical burden, however, extends beyond pulmonary impairment [2, 3]. Altered respiratory mechanics, skeletal and respiratory muscle dysfunction, exercise intolerance, dyspnea, and psychological distress interact to restrict daily functioning [4, 5]. Expiratory flow limitation and loss of elastic recoil promote lung hyperinflation, placing the diaphragm at a mechanical disadvantage and increasing the load on the inspiratory muscles [6]. These changes impair ventilatory efficiency and contribute to exertional dyspnea, reduced walking capacity, and limitations in daily activities [7, 8].

Pulmonary rehabilitation is central to the non-pharmacological management of COPD and commonly combines exercise training with education, breathing strategies, and psychosocial support [9]. Aerobic exercise (AE) improves functional capacity, cardiovascular conditioning, and tolerance to exertional symptoms [10], whereas inspiratory muscle training (IMT) specifically targets the ventilatory muscles and can improve maximal inspiratory pressure, dyspnea, exercise tolerance, and health-related quality of life [11]. Because AE and IMT address partly distinct components of exercise limitation, their combination may provide complementary systemic and respiratory benefits. However, few controlled studies have directly compared IMT, AE, combined IMT and AE (IMT_AE_), placebo IMT, and usual care within a single experimental design [12].

Whether training-related improvements persist after supervised exercise ends is also clinically relevant [13]. Detraining—the partial or complete loss of training-induced adaptations after exercise cessation—is well recognized in exercise physiology, yet its early course following respiratory muscle training in COPD remains poorly characterized [14, 15]. Pulmonary rehabilitation programs typically extend over 8–12 weeks [13], leaving uncertainty about whether meaningful physiological, functional, and patient-reported changes can emerge after a shorter supervised intervention and how rapidly these responses attenuate once training ceases. Examining this early post-intervention period may reveal which adaptations are initially retained and help inform subsequent maintenance strategies.

Sex may further influence the clinical presentation of COPD and responses to rehabilitation [16, 17]. At comparable levels of airflow limitation, women often report greater dyspnea and symptom burden than men, potentially reflecting differences in airway size, lung volumes, respiratory mechanics, hormonal influences, and respiratory muscle characteristics [16, 18]. Despite these differences, women remain underrepresented in exercise-based COPD intervention research [16, 18].

We therefore compared the effects of four weeks of IMT, AE, IMT_AE_, placebo IMT, and usual care on respiratory outcomes, functional capacity, dyspnea, and psychological well-being in women with stable COPD. We also examined the early persistence and attenuation of intervention-induced changes at 7 and 14 days after training cessation. We hypothesized that IMT, alone or combined with AE, would improve respiratory muscle function, walking capacity, dyspnea, and well-being, with the magnitude and short-term persistence of these responses differing among interventions.

## Methods

### Experimental design and ethical approval

This single-center, five-arm, parallel-group, partially blinded randomized controlled trial was conducted at Hitit University Çorum Erol Olçok Training and Research Hospital. The protocol was approved before enrollment by the Hitit University Clinical Research Ethics Committee (No. 2026-08; March 26, 2026), and all participants provided written informed consent. The study complied with the Declaration of Helsinki and is reported according to CONSORT. The trial was retrospectively registered at ClinicalTrials.gov (NCT07604961; submitted May 17 and posted May 22, 2026). Although registration was retrospective, the eligibility criteria, interventions, assessment schedule, and outcomes had been approved before enrollment and were not changed after the trial began.

Enrollment and testing ran from April 6 to May 15, 2026. Participants received written and verbal instructions and were familiarized with the testing and training procedures 3 days before baseline assessment. The intervention began after baseline testing and continued for 4 weeks; assessments were repeated immediately after the intervention and 7 and 14 days after training cessation. The prespecified primary outcome was the baseline-to-post-intervention change in maximal inspiratory pressure (MIP). Secondary outcomes were maximal expiratory pressure (MEP), peak inspiratory flow rate (PIFR), inspiratory volume (IV), forced vital capacity (FVC), forced expiratory volume in 1 s (FEV₁), FEV₁/FVC, 6-min walk test (6MWT) distance, post-6MWT Borg dyspnea, and WHO-5 well-being.

**Fig. 1:**
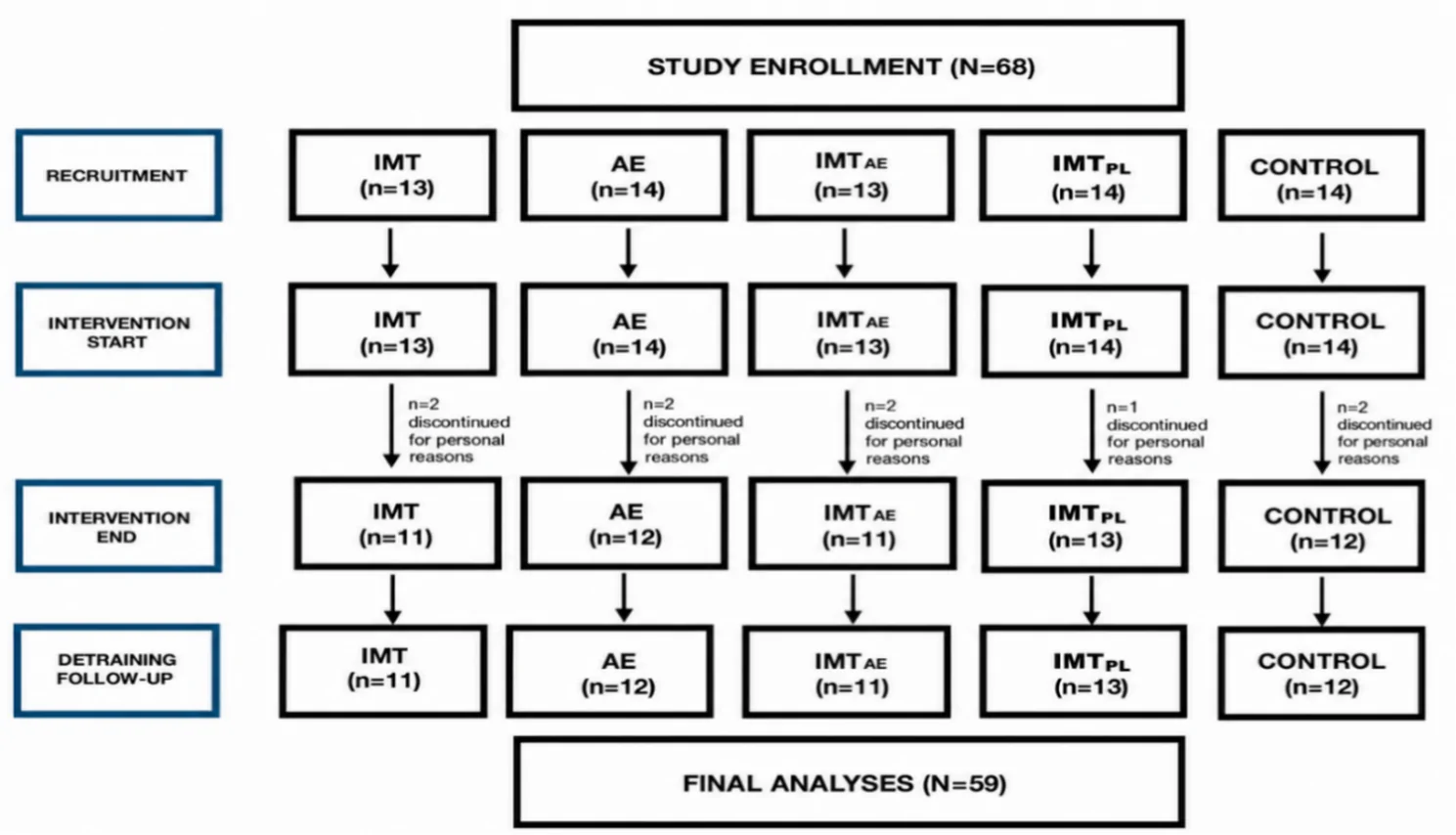
Participant flow diagram

Before each assessment, participants avoided long-distance travel, strenuous activity, alcohol, and caffeine for 24 h and consumed no food, other than water, for 3 h. Compliance was confirmed verbally. A multidisciplinary team of physicians (MD), exercise scientists (PhD), and physiotherapists (PhD) conducted the assessments, with the same researchers testing all participants to limit inter-rater variation. Participant flow is shown in Fig. 1.

### Participants

Women aged 48–65 years with clinically stable GOLD stage I–II COPD were recruited from the hospital’s Chest Diseases Outpatient Clinic. Eligibility required a documented diagnosis of stable GOLD stage I or II COPD, no exacerbation during the preceding 6 weeks, routine outpatient follow-up, abstinence from cigarettes, hookah, electronic cigarettes, and other tobacco-derived products for at least 2 months, ability to communicate in Turkish, and no regular moderate- or high-intensity exercise on ≥ 3 days/week during the preceding 3 months. Exclusion criteria were uncontrolled cardiovascular or systemic disease, severe neurological impairment, active rheumatologic disease, severe orthopedic limitation, long-term home oxygen therapy, inability to read or understand Turkish, previous IMT, respiratory muscle training, or pulmonary rehabilitation, and structured professional or recreational exercise training.

The sample size was calculated for MIP in G*Power 3.1.9.6 (Kiel, Germany) using repeated-measures ANOVA with a within–between interaction, five groups, and four assessments. With f = 0.25, α = 0.05, 80% power, a repeated-measures correlation of 0.50, and a nonsphericity correction of 0.75, 46 participants were required. The medium effect reflected the variable, generally moderate MIP responses reported after exercise-based interventions and IMT in COPD; the four-assessment calculation incorporated the primary baseline-to-post contrast and the two detraining assessments. To accommodate allocation and attrition, 68 participants were randomized; 59 completed all assessments and entered the per-protocol analysis (age, 57.58 ± 4.09 years; height, 159.69 ± 5.17 cm; body mass, 67.34 ± 6.60 kg; body mass index, 26.40 ± 2.14 kg/m²).

### Randomization and blinding

Randomization occurred after baseline testing and was stratified by MIP. Participants were ranked by ascending MIP, and consecutive sets of five formed blocks. Within each block, a computer-generated list assigned one participant to each condition (control, IMT, aerobic exercise [AE], combined IMT and AE [IMT_AE_], or placebo IMT) in a 1:1:1:1:1 ratio. After 13 complete blocks (*n* = 65), the final three participants were allocated by the same procedure to AE, control, and placebo IMT. An independent statistician generated and retained the sequence and had no role in recruitment, assessment, supervision, or analysis. The clinical team enrolled participants, and assignments were released only after baseline measurements.

In the IMT and placebo IMT conditions, participants and session supervisors were blinded to condition and resistance. Active and placebo sessions were intermingled; before each session, the principal investigator preset the devices and concealed the adjustment mechanism. Blinding was not feasible for AE or IMT_AE_. Post-intervention and detraining assessors were blinded except where assignment to the overt AE and IMT_AE_ conditions could not be concealed. Group codes remained masked from the data analysts until the prespecified analyses were complete. Blinding integrity was not formally assessed.

### Training protocols

The intervention protocols implemented for each group are described below.

#### Control condition

Control participants continued usual medical care and daily activities without supervised IMT or AE and were asked not to begin structured exercise during the 4-week intervention.

#### Inspiratory muscle training

Supervised IMT was performed once daily on Monday, Wednesday, and Friday for 4 weeks using a POWERbreathe Classic Light Resistance device (POWERbreathe International Ltd., UK). The active IMT and IMT_AE_ groups completed two sets of 30 breaths at 40% of individual MIP [19]. The IMT group performed the sets consecutively; IMT_AE_ performed one set before and one after AE. Placebo IMT followed the same schedule and procedures at 15% MIP [20]. This load preserved procedural similarity while minimizing the intended stimulus but was regarded as a low-load comparator rather than physiologically inert.

Training was completed seated under standard laboratory conditions. Participants performed strong, complete inspirations through the device followed by relaxed expirations, with brief between-set rest when required. Loads were recalibrated each Monday from the most recent MIP value. Supervisors recorded attendance and prescribed repetitions at every session.

#### Aerobic exercise protocol

The AE and IMT_AE_ groups completed supervised treadmill walking on Monday, Wednesday, and Friday in a well-ventilated indoor gym. Participants walked briskly without running at 50%–65% of age-predicted maximal heart rate (220 − age) [21, 22] and a Borg rating of perceived exertion of 4–6. Heart rate was monitored continuously with a Polar Verity Sense optical sensor (Polar Elektro H.K. Ltd., Malaysia), and treadmill speed was adjusted to maintain the target range [23].

Each 40-min session comprised a 5-min warm-up of low-intensity walking and breathing exercises, 30 min of treadmill walking, and a 5-min walking cool-down. Speed was increased by approximately 5% each week when tolerated. Heart rate, dyspnea, and oxygen saturation were monitored throughout; SpO₂ was measured with a Beurer PO 80 pulse oximeter (Beurer Medical, Germany). Workload was reduced when monitoring variables exceeded the predefined limits. Exercise was terminated if SpO₂ fell below 85%, Borg dyspnea exceeded 6, cardiac symptoms occurred, or symptoms persisted after workload reduction. A pulmonologist, sports scientist, and physiotherapist experienced in exercise interventions supervised all sessions.

### Concomitant pharmacological treatment

Participants continued their prescribed COPD treatment throughout the intervention and detraining periods, and pharmacological therapy was not modified as part of the study protocol. Baseline maintenance inhaled treatment was documented according to the regimen in use at study entry and classified as no maintenance inhaled therapy, long-acting β₂-agonist monotherapy, or combined long-acting β₂-agonist/long-acting muscarinic antagonist therapy. Participants were asked at each assessment visit about COPD exacerbations and any changes in prescribed treatment since the preceding visit. No bronchodilator was administered specifically for study testing procedures.

### Detraining period

During the two-week detraining period, participants did not receive supervised IMT or AE. They were instructed to maintain their usual daily activities and not to start any new structured exercise program. Follow-up assessments were performed on days 7 and 14 after the post-intervention measurement using the same testing procedures applied at baseline and post-test.

### Testing protocols

#### Respiratory assessments

MIP and MEP were measured with a Micro Medical/CareFusion MicroRPM (Micro Medical/CareFusion, Kent, UK) [19]. Participants were seated, wore nose clips, and maintained a tight mouthpiece seal. For MEP, they inhaled to total lung capacity and then expired rapidly and forcefully; for MIP, they exhaled to residual volume and immediately performed a maximal inspiratory effort. Each maneuver was repeated at least three times and accepted when successive values differed by < 20%; the highest value was analyzed. Standardized verbal encouragement was provided in accordance with ATS/ERS recommendations [24].

PIFR and IV were measured simultaneously in the single-breath mode of a POWERbreathe K5 (POWERbreathe International Ltd., Warwickshire, UK) [25]. Participants were seated with nose clips, exhaled to residual volume, and then inhaled forcefully for at least 1 s. Three trials were completed, and the highest PIFR and IV values were analyzed.

Spirometry was performed with a calibrated Spirometrics 3100 (Beijing M&B Electronic Instruments Co., Ltd., Beijing, China) according to ATS/ERS standards [26]. After the procedure was explained and demonstrated, seated participants wore nose clips, maintained a tight mouthpiece seal, inhaled to total lung capacity, and then exhaled as rapidly, forcefully, and completely as possible. Maneuvers with coughing, leakage, hesitation, or premature termination were rejected. At least three acceptable, reproducible trials were obtained, with additional attempts when required; the highest acceptable FVC and FEV₁ were used, and FEV₁/FVC was recorded. Testing was performed under standardized laboratory conditions and, where possible, at a similar time of day. Regular COPD medication remained unchanged.

#### Dyspnea assessment

Dyspnea was assessed using the Modified Borg Scale (0–10) immediately before and after the 6MWT [27]. The post-test score was the primary dyspnea outcome. Participants rated breathlessness related specifically to respiratory effort, excluding muscular fatigue and general tiredness. All assessments were administered by the same researcher in a quiet, distraction-free environment, and the reported score was analyzed as a subjective measure of dyspnea severity.

#### Psychological well-being assessment

The WHO-5 comprises five positively worded items scored from 0 to 5. The raw total (0–25) was multiplied by four to give a 0–100 score, with higher scores indicating better well-being. It was administered under standardized conditions at each assessment. The Turkish version has good validity, reliability, and internal consistency (Cronbach’s α = 0.81) [28].

#### Six-minute walk test

Functional exercise capacity was assessed using the 6MWT according to American Thoracic Society guidelines [29]. Participants were instructed to walk as far as possible without running for 6 min along a straight, flat, obstacle-free 40-m corridor. Brief rests were permitted for fatigue, breathlessness, or chest discomfort, but the timer continued. Standardized encouragement was provided every minute in a consistent tone, and the total distance completed between the turning points was recorded in meters.

### Adherence and safety

The supervised groups were scheduled for 12 sessions. Attendance was verified by supervisors and expressed as a percentage of prescribed sessions; missing ≥ 3 sessions precluded inclusion in the per-protocol analysis. Adverse events and clinically relevant symptoms—including excessive dyspnea, dizziness, chest discomfort, oxygen desaturation, musculoskeletal pain, and COPD exacerbation—were recorded from direct observation or participant report rather than a standardized questionnaire. Heart rate, perceived exertion, dyspnea, and SpO₂ were monitored during AE and IMT_AE_ according to the prespecified safety criteria.

### Statistical analysis

Statistical analyses were performed using IBM SPSS Statistics 25.0; data are presented as mean ± SD. Normality and homogeneity were assessed using Shapiro–Wilk tests, Q–Q plots, and Levene’s test. Baseline groups were compared using one-way ANOVA and completers versus non-completers using Welch’s t tests. The primary analysis tested the group × time interaction for MIP from baseline to post-intervention using a 5-group × 2-time mixed-design repeated-measures analysis of variance. Secondary longitudinal analyses used 5-group × 4-time mixed-design repeated-measures analyses of variance for MIP and each secondary outcome across baseline, post-intervention, DT1, and DT2. For the four-time analyses, Mauchly’s test assessed sphericity, with Greenhouse–Geisser correction applied when required [30]. Significant effects were followed by Bonferroni-adjusted comparisons. All completers had complete data at all four assessments. Baseline-to-post changes in each active group were additionally compared with control and placebo IMT using Welch’s t tests and reported as mean differences with 95% CIs; these unadjusted comparisons were exploratory. Effect sizes were ηp², Cohen’s dz, and Cohen’s d for omnibus, within-group, and between-group effects, respectively. Sensitivity analyses used random-intercept linear mixed models with fixed group, time, and group × time effects; interactions were evaluated using likelihood-ratio tests comparing full and reduced models, with Holm adjustment across outcomes. As all nine withdrawals lacked post-baseline data, neither intention-to-treat analysis nor post-withdrawal imputation was performed. Two-sided *p* < 0.05 indicated statistical significance.

## Results

Of the 68 randomized participants, 59 completed all assessments and were included in the per-protocol analysis (86.8%): 11/13 in IMT (84.6%), 12/14 in AE (85.7%), 11/13 in IMT_AE_ (84.6%), 12/14 in CONT (85.7%), and 13/14 in IMT_PL_ (92.9%). Nine participants withdrew voluntarily for personal reasons (two each from IMT, AE, IMT_AE_, and CONT and one from IMT_PL_). No intervention-related adverse events, COPD exacerbations, or treatment changes occurred during the intervention or detraining periods.

Among the 47 completers assigned to supervised interventions, 547/564 prescribed sessions were completed (97.0%): 95.5% in IMT, 98.6% in AE, 97.0% in IMT_AE_, and 96.8% in IMT_PL_. All completed at least 10 of 12 sessions; therefore, no completer was excluded for insufficient attendance (Supplementary Table S6). At baseline, 47/59 participants (79.7%) received no maintenance inhaled therapy, 11 (18.6%) received LABA/LAMA, and one (1.7%) received LABA monotherapy; none received LAMA monotherapy or an ICS-containing regimen (Supplementary Table S5). Fifty-five participants had GOLD stage I and four had GOLD stage II COPD.

The nine non-completers had no post-intervention or detraining data. Baseline MIP was similar in completers and non-completers (60.25 ± 9.02 vs. 60.22 ± 9.54 cmH₂O), with no evident differences in age, body mass, BMI, respiratory muscle strength, inspiratory performance, pulmonary function, 6MWT distance, or mMRC dyspnea (Supplementary Table S3). Baseline characteristics were also comparable across study groups (Table 1).

**Table 1:** Baseline characteristics of the per-protocol sample by study group

Variable	IMT	AE	IMT_AE_	CONT	IMT_PL_	*p*	η²
Age (years)	56.82 ± 3.95	58.00 ± 3.52	57.55 ± 4.59	57.92 ± 4.21	57.54 ± 4.70	0.967	0.010
Height (cm)	159.65 ± 4.14	160.23 ± 5.84	159.95 ± 6.13	158.93 ± 5.67	159.69 ± 4.66	0.982	0.007
Body mass (kg)	68.05 ± 7.41	67.56 ± 7.87	70.25 ± 7.16	66.53 ± 3.29	64.82 ± 6.30	0.367	0.075
MIP (cmH₂O)	62.00 ± 7.99	61.17 ± 10.00	59.55 ± 9.77	59.08 ± 9.01	59.62 ± 9.41	0.934	0.015
MEP (cmH₂O)	75.55 ± 5.91	72.25 ± 7.78	73.64 ± 7.38	72.67 ± 6.58	73.08 ± 6.68	0.812	0.028
PIFR (L/s)	3.76 ± 0.25	3.69 ± 0.29	3.70 ± 0.23	3.68 ± 0.32	3.70 ± 0.30	0.956	0.012
IV (L)	2.35 ± 0.12	2.40 ± 0.19	2.34 ± 0.18	2.24 ± 0.24	2.28 ± 0.22	0.296	0.086
6MWT Distance (m)	446.27 ± 20.97	432.33 ± 27.97	437.00 ± 25.48	440.83 ± 19.66	441.38 ± 22.86	0.689	0.040
FVC (L)	3.40 ± 0.29	3.30 ± 0.34	3.32 ± 0.29	3.28 ± 0.39	3.32 ± 0.35	0.940	0.014
FEV₁ (L)	2.12 ± 0.22	2.05 ± 0.25	2.04 ± 0.19	2.01 ± 0.28	2.05 ± 0.26	0.850	0.025
FEV₁/FVC	0.62 ± 0.02	0.62 ± 0.02	0.61 ± 0.02	0.61 ± 0.02	0.61 ± 0.02	0.453	0.064
Modified BORG dyspnea score	2.64 ± 0.67	2.67 ± 0.78	3.18 ± 0.60	2.50 ± 0.52	2.62 ± 0.65	0.129	0.122
WHO-5 Well-Being Index score	61.45 ± 7.85	58.33 ± 10.16	55.27 ± 8.91	59.67 ± 7.71	60.31 ± 7.74	0.489	0.060

Data are mean ± standard deviation. *p* values are from one-way analysis of variance; η² denotes eta squared. Higher Borg scores indicate greater dyspnea

The primary baseline-to-post group × time interaction for MIP was significant (F(4, 54) = 28.969, *p* < 0.001, ηp² = 0.682). In the secondary four-time analyses, significant time effects were observed for all outcomes except FEV₁/FVC (F = 2.720, *p* = 0.103, ηp² = 0.048). Significant group × time interactions were observed for MIP, MEP, PIFR, IV, 6MWT distance, FVC, FEV₁, post-exercise Borg dyspnea, and WHO-5 (all *p* < 0.001), but not for FEV₁/FVC (F = 1.211, *p* = 0.316, ηp² = 0.082; Table 2).

**Table 2:** Four-time mixed repeated-measures ANOVA results

Variable	Time			Group × Time		
	F	*p*	ηp²	F	*p*	ηp²
MIP (cmH₂O)	116.886	< 0.001	0.684	20.925	< 0.001	0.608
MEP (cmH₂O)	20.459	< 0.001	0.275	10.355	< 0.001	0.434
PIFR (L/s)	326.382	< 0.001	0.858	78.836	< 0.001	0.854
IV (L)	111.527	< 0.001	0.674	23.590	< 0.001	0.636
6MWT Distance (m)	164.132	< 0.001	0.752	43.197	< 0.001	0.762
FVC (L)	28.045	< 0.001	0.342	11.045	< 0.001	0.450
FEV₁ (L)	15.833	< 0.001	0.227	6.531	< 0.001	0.326
FEV₁/FVC Ratio	2.720	0.103	0.048	1.211	0.316	0.082
Modified BORG Dyspnea Score	44.802	< 0.001	0.453	9.490	< 0.001	0.413
WHO-5 Well-Being Index Score	69.690	< 0.001	0.563	15.582	< 0.001	0.536

Greenhouse–Geisser-corrected *p* values are reported when sphericity was violated. ηp², partial eta squared

Exploratory linear mixed-effects models produced the same pattern after Holm adjustment: interactions remained significant for all outcomes except FEV₁/FVC (χ² = 15.207, df = 12, adjusted *p* = 0.230; Supplementary Table S4).

MIP increased from baseline to post-intervention by 5.09 cmH₂O in IMT, 2.50 cmH₂O in AE, 9.45 cmH₂O in IMT_AE_, and 2.31 cmH₂O in IMT_PL_, with no change in CONT (Table 3 and Supplementary Table S1). Values remained above baseline at DT1 in IMT and at both DT1 and DT2 in AE, IMT_AE_, and IMT_PL_, although each group showed reductions after training ceased.

**Table 3:** Changes in respiratory muscle and inspiratory performance outcomes

Variable	Group	Pre	Post	DT1	DT2	Within- group F	*p*	ηp²
MIP (cmH₂O)	IMT	62.00 ± 7.99	67.09 ± 8.86	65.55 ± 8.65	64.09 ± 8.78	19.255	< 0.001	0.658
	AE	61.17 ± 10.00	63.67 ± 9.93	63.17 ± 9.92	62.42 ± 9.91	17.543	0.001	0.615
	IMT_AE_	59.55 ± 9.77	69.00 ± 9.71	66.45 ± 9.35	65.55 ± 9.67	67.389	< 0.001	0.871
	CONT	59.08 ± 9.01	58.92 ± 8.83	59.00 ± 8.99	59.00 ± 8.79	0.133	0.778	0.012
	IMT_PL_	59.62 ± 9.41	61.92 ± 9.78	61.23 ± 9.61	60.92 ± 9.63	30.095	< 0.001	0.715
MEP (cmH₂O)	IMT	75.55 ± 5.91	77.36 ± 6.42	76.36 ± 6.28	75.91 ± 6.33	7.345	0.011	0.423
	AE	72.25 ± 7.78	73.17 ± 7.78	72.83 ± 7.79	72.58 ± 7.75	10.672	0.002	0.492
	IMT_AE_	73.64 ± 7.38	76.82 ± 6.87	75.91 ± 7.63	74.73 ± 7.54	23.036	< 0.001	0.697
	CONT	72.67 ± 6.58	72.17 ± 6.63	72.42 ± 6.44	72.25 ± 6.57	1.638	0.226	0.130
	IMT_PL_	73.08 ± 6.68	72.46 ± 6.68	72.62 ± 6.58	72.62 ± 6.49	2.512	0.074	0.173
PIFR (L/s)	IMT	3.76 ± 0.25	4.09 ± 0.23	3.99 ± 0.23	3.89 ± 0.23	394.861	< 0.001	0.975
	AE	3.69 ± 0.29	3.79 ± 0.29	3.77 ± 0.29	3.69 ± 0.29	62.333	< 0.001	0.850
	IMT_AE_	3.70 ± 0.23	4.16 ± 0.23	4.01 ± 0.21	3.91 ± 0.21	507.407	< 0.001	0.981
	CONT	3.68 ± 0.32	3.67 ± 0.31	3.67 ± 0.31	3.62 ± 0.28	7.563	0.007	0.407
	IMT_PL_	3.70 ± 0.30	3.72 ± 0.25	3.70 ± 0.24	3.68 ± 0.28	0.787	0.509	0.062
IV (L)	IMT	2.35 ± 0.12	2.50 ± 0.21	2.48 ± 0.21	2.46 ± 0.20	10.932	0.006	0.522
	AE	2.40 ± 0.19	2.69 ± 0.24	2.60 ± 0.21	2.54 ± 0.20	54.135	< 0.001	0.831
	IMT_AE_	2.34 ± 0.18	2.67 ± 0.23	2.56 ± 0.20	2.47 ± 0.19	69.364	< 0.001	0.874
	CONT	2.24 ± 0.24	2.25 ± 0.23	2.25 ± 0.23	2.24 ± 0.23	0.171	0.690	0.015
	IMT_PL_	2.28 ± 0.22	2.27 ± 0.22	2.27 ± 0.22	2.27 ± 0.22	1.693	0.216	0.124

Data are mean ± standard deviation. Pre, baseline; Post, post-intervention; DT1 and DT2, 7 and 14 days after intervention cessation, respectively. F, p, and ηp² represent within-group time effects; ηp² denotes partial eta squared

MEP increased by 1.82 cmH₂O in IMT, 0.92 cmH₂O in AE, and 3.18 cmH₂O in IMT_AE_, but not in CONT or IMT_PL_. The increase remained significant at DT1 in AE and IMT_AE_, with reductions during detraining, particularly by DT2.

PIFR increased by 0.33 L/s in IMT, 0.10 L/s in AE, and 0.46 L/s in IMT_AE_ and remained above baseline through DT2 in IMT and IMT_AE_. At post-intervention, PIFR was higher in IMT than in CONT and IMTPL and higher in IMT_AE_ than in AE, CONT, and IMT_PL_. Only the IMT_AE_–IMT_PL_ difference remained at DT1, and no between-group differences remained at DT2. IV increased by 0.15 L in IMT, 0.29 L in AE, and 0.33 L in IMT_AE_. Gains persisted through DT2 in AE and IMT_AE_, whereas the IMT gain had declined by DT2. At post-intervention and DT1, IV was higher in AE and IMT_AE_ than in CONT and IMT_PL_; at DT2, only AE remained higher than both comparators (Table 3 and Supplementary Tables S1 and S2). Complete Bonferroni-adjusted pairwise comparisons and corresponding Cohen’s d and d_z values are reported in Supplementary Tables S1 and S2.

FVC increased in AE and IMT_AE_ and remained above baseline at post-intervention, DT1, and DT2, despite reductions after training ceased (Table 4 and Supplementary Table S1). IMT showed no adjusted baseline-to-post increase in FVC, although values decreased from post-intervention at DT1 and DT2.

**Table 4:** Changes in pulmonary function and dyspnea outcomes

Variable	Group	Pre	Post	DT1	DT2	Within-group F	*p*	ηp²
FVC (L)	IMT	3.40 ± 0.29	3.45 ± 0.29	3.40 ± 0.28	3.39 ± 0.29	5.170	0.044	0.341
	AE	3.30 ± 0.34	3.38 ± 0.35	3.36 ± 0.34	3.34 ± 0.34	19.160	0.001	0.635
	IMT_AE_	3.32 ± 0.29	3.44 ± 0.29	3.41 ± 0.29	3.39 ± 0.29	17.626	0.002	0.638
	CONT	3.28 ± 0.39	3.26 ± 0.39	3.27 ± 0.38	3.27 ± 0.39	10.506	0.005	0.489
	IMT_PL_	3.32 ± 0.35	3.31 ± 0.35	3.31 ± 0.35	3.32 ± 0.35	2.215	0.158	0.156
FEV₁ (L)	IMT	2.12 ± 0.22	2.14 ± 0.22	2.14 ± 0.21	2.13 ± 0.21	0.284	0.607	0.028
	AE	2.05 ± 0.25	2.11 ± 0.28	2.09 ± 0.27	2.08 ± 0.26	8.456	0.014	0.435
	IMT_AE_	2.04 ± 0.19	2.16 ± 0.21	2.13 ± 0.21	2.10 ± 0.20	19.634	0.001	0.663
	CONT	2.01 ± 0.28	2.00 ± 0.28	2.00 ± 0.28	2.00 ± 0.28	4.904	0.040	0.308
	IMT_PL_	2.05 ± 0.26	2.04 ± 0.26	2.04 ± 0.26	2.04 ± 0.26	1.406	0.264	0.105
FEV₁/FVC ratio	IMT	0.62 ± 0.02	0.62 ± 0.04	0.63 ± 0.03	0.63 ± 0.02	0.498	0.504	0.047
	AE	0.62 ± 0.02	0.62 ± 0.03	0.62 ± 0.03	0.62 ± 0.02	0.213	0.662	0.019
	IMT_AE_	0.61 ± 0.02	0.63 ± 0.03	0.62 ± 0.02	0.62 ± 0.02	12.729	0.004	0.560
	CONT	0.61 ± 0.02	0.61 ± 0.02	0.61 ± 0.02	0.61 ± 0.02	1.464	0.255	0.117
	IMT_PL_	0.61 ± 0.02	0.61 ± 0.02	0.62 ± 0.02	0.61 ± 0.02	1.009	0.352	0.078
Modified BORG Dyspnea Score	IMT	2.64 ± 0.67	2.00 ± 0.45	2.09 ± 0.30	2.36 ± 0.67	8.333	< 0.001	0.455
	AE	2.67 ± 0.78	2.08 ± 0.67	2.33 ± 0.49	2.67 ± 0.78	9.497	0.002	0.463
	IMT_AE_	3.18 ± 0.60	2.09 ± 0.54	2.36 ± 0.50	2.91 ± 0.54	29.032	< 0.001	0.744
	CONT	2.50 ± 0.52	2.50 ± 0.52	2.50 ± 0.52	2.50 ± 0.52	-	-	-
	IMT_PL_	2.62 ± 0.65	2.62 ± 0.65	2.62 ± 0.65	2.62 ± 0.65	-	-	-

Data are mean ± standard deviation. Pre, baseline; Post, post-intervention; DT1 and DT2, 7 and 14 days after intervention cessation, respectively. F, p, and ηp² represent within-group time effects; ηp² denotes partial eta squared. Borg tests were not estimable in CONT or IMT_PL_ because no within-participant variation occurred; higher scores indicate greater dyspnea

FEV₁ increased only in IMT_AE_ (+ 0.11 L) and remained above baseline at both detraining assessments, with reductions from post-intervention. FEV₁/FVC showed no group × time interaction; within IMT_AE_, it increased at post-intervention and DT1 and subsequently declined.

Post-exercise Borg dyspnea decreased from baseline to post-intervention by 0.64 points in IMT, 0.58 points in AE, and 1.09 points in IMT_AE_ (Table 4 and Supplementary Table S1). The reduction persisted at DT1 in IMT and IMT_AE_, but not at DT2 in any group. Scores increased from post-intervention to DT2 in AE and IMT_AE_ and from DT1 to DT2 in IMT_AE_. Bonferroni-adjusted between-group comparisons were not significant at post-intervention (Supplementary Table S2).

The 6MWT distance increased by 5.09 m in IMT, 9.67 m in AE, and 16.09 m in IMT_AE_ and remained above baseline at DT1 and DT2 in all three groups (Table 5 and Supplementary Table S1). Distance declined from post-intervention at both detraining assessments in AE and IMT_AE_ and by DT2 in IMT. CONT and IMT_PL_ showed no change. WHO-5 increased by 5.82 points in IMT, 5.33 points in AE, and 9.45 points in IMT_AE_. Improvements remained at DT1 in all active groups and at DT2 only in IMT_AE_; scores declined from post-intervention to DT2 and from DT1 to DT2 in all three groups. No changes occurred in CONT or IMT_PL_.

**Table 5:** Functional and secondary clinical outcomes

Variable	Group	Pre	Post	DT1	DT2	Within-group F	*p*	ηp²
6MWT Distance (m)	IMT	446.27 ± 20.97	451.36 ± 22.20	450.18 ± 21.76	449.55 ± 21.79	22.575	< 0.001	0.693
	AE	432.33 ± 27.97	442.00 ± 26.92	439.17 ± 27.12	436.75 ± 27.36	75.816	< 0.001	0.873
	IMT_AE_	437.00 ± 25.48	453.09 ± 27.10	447.45 ± 26.39	443.27 ± 25.78	127.942	< 0.001	0.928
	CONT	440.83 ± 19.66	440.75 ± 20.01	440.75 ± 19.85	440.83 ± 19.78	0.029	0.904	0.003
	IMT_PL_	441.38 ± 22.86	441.00 ± 24.03	441.15 ± 23.66	441.00 ± 23.16	0.212	0.682	0.017
WHO-5 Well-Being Index score	IMT	61.45 ± 7.85	67.27 ± 8.16	65.82 ± 7.45	63.27 ± 8.16	35.941	< 0.001	0.782
	AE	58.33 ± 10.16	63.67 ± 10.01	62.00 ± 10.16	59.67 ± 10.71	25.362	< 0.001	0.697
	IMT_AE_	55.27 ± 8.91	64.73 ± 9.43	61.45 ± 9.51	58.55 ± 8.25	87.059	< 0.001	0.897
	CONT	59.67 ± 7.71	60.00 ± 8.18	60.00 ± 8.86	59.33 ± 7.97	0.387	0.763	0.034
	IMT_PL_	60.31 ± 7.74	59.38 ± 8.77	59.08 ± 9.11	59.69 ± 7.74	1.228	0.314	0.093

Data are mean ± standard deviation. Pre, baseline; Post, post-intervention; DT1 and DT2, 7 and 14 days after intervention cessation, respectively. F, p, and ηp² represent within-group time effects; ηp² denotes partial eta squared

Exploratory baseline-to-post change-score comparisons are shown in Tables 6, 7, 8, 9. For MIP, IMT_AE_ improved more than CONT (mean difference, 9.62 cmH₂O; 95% CI, 7.13–12.11; *p* < 0.001) and IMT_PL_ (7.15 cmH₂O; 95% CI, 4.67–9.63; *p* < 0.001). IMT also exceeded both comparators, whereas AE exceeded CONT but not IMT_PL_. All active groups improved MEP more than both comparators, with the largest differences for IMT_AE_ (Table 6).

**Table 6:** Baseline-to-post between-group differences in respiratory outcomes

Outcome	Comparison	Mean difference	95% CI	*p* value
MIP (cmH₂O)	IMT_AE_ vs. CONT	9.62	7.13 to 12.11	< 0.001
	IMT_AE_ vs. IMT_PL_	7.15	4.67 to 9.63	< 0.001
	IMT vs. CONT	5.26	3.44 to 7.07	< 0.001
	IMT vs. IMT_PL_	2.78	0.99 to 4.58	0.005
	AE vs. CONT	2.67	1.14 to 4.19	0.002
	AE vs. IMT_PL_	0.19	−1.31 to 1.69	0.792
MEP (cmH₂O)	IMT_AE_ vs. CONT	3.68	2.18 to 5.18	< 0.001
	IMT_AE_ vs. IMT_PL_	3.80	2.34 to 5.25	< 0.001
	IMT vs. CONT	2.32	1.10 to 3.53	< 0.001
	IMT vs. IMT_PL_	2.43	1.28 to 3.59	< 0.001
	AE vs. CONT	1.42	0.46 to 2.38	0.006
	AE vs. IMT_PL_	1.53	0.66 to 2.40	0.001

Mean difference is the baseline-to-post change in the first group minus that in the second; positive values indicate a larger increase in the first group. Comparisons are exploratory; two-sided Welch *p* values are unadjusted for multiplicity

**Table 7:** Baseline-to-post between-group differences in inspiratory performance

Outcome	Comparison	Mean difference	95% CI	*p* value
PIFR (L/s)	IMT_AE_ vs. CONT	0.47	0.44 to 0.51	< 0.001
	IMT_AE_ vs. IMT_PL_	0.45	0.40 to 0.50	< 0.001
	IMT vs. CONT	0.34	0.30 to 0.37	< 0.001
	IMT vs. IMT_PL_	0.31	0.26 to 0.36	< 0.001
	AE vs. CONT	0.11	0.09 to 0.13	< 0.001
	AE vs. IMT_PL_	0.08	0.04 to 0.13	< 0.001
IV (L)	IMT_AE_ vs. CONT	0.33	0.23 to 0.42	< 0.001
	IMT_AE_ vs. IMT_PL_	0.34	0.25 to 0.43	< 0.001
	IMT vs. CONT	0.14	0.05 to 0.24	0.006
	IMT vs. IMT_PL_	0.16	0.07 to 0.25	0.003
	AE vs. CONT	0.28	0.19 to 0.37	< 0.001
	AE vs. IMT_PL_	0.30	0.21 to 0.38	< 0.001

Mean difference is the baseline-to-post change in the first group minus that in the second; positive values indicate a larger increase in the first group. Comparisons are exploratory; two-sided Welch p values are unadjusted for multiplicity

**Table 8:** Baseline-to-post between-group differences in pulmonary function

Outcome	Comparison	Mean difference	95% CI	*p* value
FVC (L)	IMT_AE_ vs. CONT	0.14	0.07 to 0.20	< 0.001
	IMT_AE_ vs. IMT_PL_	0.13	0.07 to 0.19	0.001
	IMT vs. CONT	0.07	0.02 to 0.12	0.011
	IMT vs. IMT_PL_	0.06	0.01 to 0.11	0.022
	AE vs. CONT	0.10	0.06 to 0.14	< 0.001
	AE vs. IMT_PL_	0.09	0.05 to 0.13	< 0.001
FEV₁ (L)	IMT_AE_ vs. CONT	0.12	0.07 to 0.18	< 0.001
	IMT_AE_ vs. IMT_PL_	0.12	0.06 to 0.18	< 0.001
	IMT vs. CONT	0.03	−0.05 to 0.10	0.433
	IMT vs. IMT_PL_	0.02	−0.05 to 0.10	0.526
	AE vs. CONT	0.07	0.02 to 0.12	0.006
	AE vs. IMT_PL_	0.07	0.02 to 0.11	0.009
FEV₁/FVC	IMT_AE_ vs. CONT	0.01	0.00 to 0.02	0.009
	IMT_AE_ vs. IMT_PL_	0.01	0.00 to 0.02	0.017
	IMT vs. CONT	0.00	−0.03 to 0.02	0.718
	IMT vs. IMT_PL_	0.00	−0.03 to 0.02	0.683
	AE vs. CONT	0.00	−0.01 to 0.01	0.856
	AE vs. IMT_PL_	0.00	−0.01 to 0.01	0.945

Mean difference is the baseline-to-post change in the first group minus that in the second; positive values indicate a larger increase in the first group. Comparisons are exploratory; two-sided Welch *p* values are unadjusted for multiplicity

**Table 9:** Baseline-to-post between-group differences in functional and patient-reported outcomes

Outcome	Comparison	Mean difference	95% CI	*p* value
6MWT distance (m)	IMT_AE_ vs. CONT	16.17	12.85 to 19.50	< 0.001
	IMT_AE_ vs. IMT_PL_	16.48	12.96 to 20.00	< 0.001
	IMT vs. CONT	5.17	2.89 to 7.46	< 0.001
	IMT vs. IMT_PL_	5.48	2.87 to 8.08	< 0.001
	AE vs. CONT	9.75	7.06 to 12.44	< 0.001
	AE vs. IMT_PL_	10.05	7.10 to 13.00	< 0.001
Modified BORG Dyspnea Score	IMT_AE_ vs. CONT	−1.09	−1.29 to − 0.89	< 0.001
	IMT_AE_ vs. IMT_PL_	−1.09	−1.29 to − 0.89	< 0.001
	IMT vs. CONT	−0.64	−0.98 to − 0.30	0.002
	IMT vs. IMT_PL_	−0.64	−0.98 to − 0.30	0.002
	AE vs. CONT	−0.58	−0.91 to − 0.26	0.002
	AE vs. IMT_PL_	−0.58	−0.91 to − 0.26	0.002
WHO-5 Well-Being Index Score	IMT_AE_ vs. CONT	9.12	7.35 to 10.89	< 0.001
	IMT_AE_ vs. IMT_PL_	10.38	8.51 to 12.25	< 0.001
	IMT vs. CONT	5.48	3.68 to 7.29	< 0.001
	IMT vs. IMT_PL_	6.74	4.84 to 8.64	< 0.001
	AE vs. CONT	5.00	2.74 to 7.26	< 0.001
	AE vs. IMT_PL_	6.26	3.93 to 8.58	< 0.001

Mean difference is the baseline-to-post change in the first group minus that in the second. Positive values indicate a larger increase in the first group; for Borg dyspnea, negative mean differences indicate a larger reduction. Comparisons are exploratory; two-sided Welch *p* values are unadjusted for multiplicity

For PIFR, IMT_AE_ exceeded CONT (mean difference, 0.47 L/s; 95% CI, 0.44–0.51; *p* < 0.001) and IMT_PL_ (0.45 L/s; 95% CI, 0.40–0.50; *p* < 0.001); IMT and AE also exceeded both comparators. All active groups increased IV more than CONT and IMT_PL_, with the largest differences for IMT_AE_ and AE (Table 7).

FVC increased more in each active group than in both comparators; the largest difference was between IMT_AE_ and CONT (0.14 L; 95% CI, 0.07–0.20; *p* < 0.001). FEV₁ gains were greater for IMT_AE_ and AE than for both comparators, whereas IMT did not differ from either. Nominal FEV₁/FVC differences for IMT_AE_ versus CONT and IMT_PL_ were not interpreted because the overall interaction was not significant (Table 8).

IMT_AE_ produced the largest 6MWT gains relative to CONT (mean difference, 16.17 m; 95% CI, 12.85–19.50; *p* < 0.001) and IMT_PL_ (16.48 m; 95% CI, 12.96–20.00; *p* < 0.001); IMT and AE also exceeded both comparators. All active groups showed greater reductions in post-exercise dyspnea and greater increases in WHO-5 than CONT and IMT_PL_, with the largest differences in IMT_AE_ (Table 9).

## Discussion

The primary baseline-to-post analysis showed that MIP change differed among groups, with the largest increase after combined IMT and AE. Secondary four-time analyses showed that combined training produced the broadest response across inspiratory performance, walking capacity, post-exercise dyspnea, and well-being in women with stable GOLD stage I–II COPD. IMT primarily improved respiratory muscle and inspiratory outcomes, whereas AE produced smaller respiratory changes together with functional gains. Active interventions generally outperformed both comparators; sensitivity analysis supported the group × time effect for MIP. Several gains declined after supervised training stopped, with retention differing by outcome.

MIP increased by 9.45 cmH₂O after combined training and by 5.09 cmH₂O after IMT alone, despite baseline values of 59–62 cmH₂O and no requirement for inspiratory weakness at enrollment. This direction agrees with pooled evidence [31, 32] and original trials. Hill et al. recorded a 29% increase after eight weeks of high-intensity IMT, Beckerman et al. an increase from 71 to 90 cmH₂O after three months, and Petrovic et al. an increase from 7.75 to 9.15 kPa after eight weeks [33–35]. Charususin et al. also found greater inspiratory strength and endurance when IMT was added to rehabilitation in patients with weakness, whereas two trials by Beaumont et al. found little or no additional functional benefit when baseline strength was preserved [36–38]. The present response was smaller than gains after longer, high-intensity programs but occurred after four weeks in a relatively preserved cohort. In older women, Gökçelik et al. reported increases of 11.44% and 7.48% in mid- and posterior diaphragm thickness, respectively, after four weeks of IMT [39]. Higher PIFR and IV support improved inspiratory performance; Weiner and Weiner similarly found increases in MIP and peak inspiratory flow after eight weeks [40]. MEP also increased, although expiratory muscles were not trained directly, possibly reflecting thoracoabdominal recruitment or improved execution of maximal maneuvers.

The 2.31-cmH₂O rise in MIP after placebo IMT deserves separate consideration. The 15% MIP load reproduced the training procedure but was not physiologically inert, and repeated resisted inspirations may have improved task execution or motor coordination. Unlike active training, however, placebo IMT did not improve MEP, PIFR, IV, 6MWT distance, dyspnea, or WHO-5. The usual-care group showed no corresponding pattern. This separation between an isolated pressure change and concordant changes across respiratory, functional, and patient-reported outcomes argues against familiarization, attention, or expectation as the sole explanation for the active-treatment responses.

Combined training yielded larger changes than either modality alone, but the pattern should not be labeled synergistic because IMT_AE_ involved more total training [41]. Larson et al. found that adding IMT to cycle training increased respiratory strength and endurance without further improving peak oxygen uptake, symptoms, or performance [42]. Wang et al. likewise reported a larger MIP gain with combined than cycle training (5.20 vs. 1.32 cmH₂O), but no additional exercise, inspiratory-capacity, dyspnea, or depression benefit [43]. Charususin et al. found greater inspiratory strength and endurance and 75 s more endurance cycling, but no 6MWT difference [36]; the Beaumont trials also found no consistent added benefit for dyspnea, walking, or quality of life [37, 38]. By contrast, Hill et al. and Petrovic et al. reported parallel respiratory and exercise-related gains after targeted IMT [33, 35]. Differences in baseline weakness, training dose, and outcome selection probably account for part of this variation [44, 45]. The present profile is compatible with simultaneous ventilatory-pump loading and whole-body conditioning, but also with a greater cumulative stimulus. In sedentary women, Cicek et al. reported increases of approximately 25% and 23% in VO₂max after 12 weeks of AE and HIIT, respectively [46]. The AE arm should not be equated with comprehensive pulmonary rehabilitation, which also includes individualized assessment, education, self-management, and behavioral support [47, 48].

Combined training increased 6MWT distance by 16.09 m, compared with 9.67 m after AE and 5.09 m after IMT. Longer studies reported gains of 27 m after high-intensity IMT, 56 m after three months of IMT, and 52 m after six months of comprehensive exercise training [33, 34, 49]. Petrovic et al. improved constant-load endurance without increasing peak work rate; Charususin et al. improved endurance cycling but not 6MWT distance; and the Beaumont trials found no additional walking gain from IMT during rehabilitation [35–38]. The present 16-m gain remained below the 25–30-m minimal important difference [50, 51]. Four weeks of training and baseline distances of 432–446 m probably limited its magnitude, although the consistent between-group response indicates an early functional adaptation.

Post-exercise Borg dyspnea decreased by 1.09 points after combined training and by 0.64 and 0.58 points after IMT and AE. Hill, Beckerman, and Petrovic reported less dyspnea after IMT, with Petrovic et al. also finding reduced dynamic hyperinflation and longer constant-load endurance [33–35]. Adjunctive IMT did not further reduce post-6MWT dyspnea in the Charususin or Beaumont trials despite greater inspiratory strength [36–38]. Dyspnea physiology supports this dissociation [52, 53]: Langer et al. showed that IMT reduced relative diaphragm activation and relieved dyspnea without changing ventilation, breathing pattern, or operating lung volumes [54]. Here, lower dyspnea accompanied higher MIP, PIFR, and IV, whereas FEV₁/FVC showed no differential group × time change. Reduced inspiratory muscle loading during walking is more plausible than altered airway obstruction, although diaphragm activation and dynamic hyperinflation were not measured.

FVC and FEV₁ increased modestly, particularly after combined training, but the absence of a differential change in FEV₁/FVC argues against reversal of airflow obstruction. Trials by Hill, Larson, Petrovic, Charususin, and both Beaumont groups found respiratory muscle, endurance, or symptom improvement without a corresponding normalization of spirometry [33, 35–38, 42]. The present changes may reflect a deeper preceding inspiration, altered breathing strategy, or more forceful and reproducible spirometric performance. Ceylan et al. reported acute increases of 2.57% in FVC and 2.44% in FEV₁ after inspiratory muscle warm-up in male bodybuilders, without a between-group change in MIP [55]. Accordingly, the modest spirometric changes in the present trial should be interpreted as secondary respiratory-performance findings rather than structural modification of COPD.

WHO-5 increased by 9.45 points after combined training and by approximately 5–6 points after IMT and AE; only the combined group remained above baseline at day 14. WHO-5 assesses general well-being rather than COPD-specific health status [28], but the direction agrees with improved CRQ or SGRQ outcomes in the original trials by Hill, Beckerman, Griffiths, Güell, and Troosters [33, 34, 49, 56, 57]. Those interventions were longer and not directly comparable. In the present cohort, less exertional dyspnea and better walking performance may have improved vitality and confidence, while supervised contact may also have contributed. Pairing WHO-5 with CAT, SGRQ, or CRQ would separate general from COPD-specific benefit.

Detraining produced an early, outcome-specific loss of adaptation. MIP declined, PIFR and 6MWT distance retained part of their gains, dyspnea improvement had largely disappeared by day 14, and sustained WHO-5 improvement was confined to combined training. Weiner et al. similarly found deterioration in inspiratory performance, walking, and dyspnea over 6–12 months without IMT maintenance [58], while Griffiths, Güell, and Troosters observed partial waning after pulmonary rehabilitation [49, 56, 57]. Spencer et al. preserved 6MWT distance with either supervised or home exercise at 12 months; Ries et al. found a maintenance advantage at 12 but not 24 months; and Butler et al. observed preserved capacity only with adequate attendance [59–61]. A structured program retained benefit for two years, but not three, in Güell et al. [62]. The present day-7 and day-14 data identify the onset of attenuation, not its full course or an optimal maintenance dose.

The all-female cohort is relevant because women remain underrepresented in COPD exercise studies and may have greater symptom burden at comparable airflow limitation [16, 18]. In the 3CIA analysis, women reported more dyspnea despite higher percent-predicted FEV₁, and women in TORCH had worse dyspnea and health status and 25% more exacerbations than men [63, 64]. Cherian et al. similarly found higher dyspnea and CAT and SGRQ scores in women with mild-to-moderate COPD [18]. During matched exercise, Guenette et al. recorded higher Borg dyspnea in women, who used a greater fraction of ventilatory capacity and reached mechanical constraints at lower work rates [65]. Available rehabilitation cohorts, however, suggest that women and men can obtain similar short- and long-term benefits [66]. The present trial defines responses in women but cannot establish that they are female-specific without a male comparator.

Several limitations define the scope of inference. Four weeks of training and two weeks of follow-up capture early adaptation and attenuation, not the full magnitude or durability of rehabilitation. This modest single-center sample comprised women aged 48–65 years with stable GOLD stage I–II COPD, predominantly stage I; applicability to men, older adults, advanced disease, frequent exacerbators, or marked inspiratory weakness requires confirmation. COPD duration and pack-years were unavailable. The protocol, eligibility criteria, interventions, assessment schedule, and outcomes were approved before enrollment and remained unchanged, but public registration was retrospective. The per-protocol analysis represents completers; nine withdrawals had no post-intervention data for an observed-data intention-to-treat analysis. Baseline profiles were comparable, but attrition bias remains possible. Blinding covered IMT resistance and assessors where allocation was concealable, but not AE or combined training; blinding success was not tested. Usual care lacked matched supervised contact, leaving attention and expectation relevant to effort- and perception-dependent outcomes. Repeated testing may have influenced MIP, PIFR, and 6MWT performance, although controls showed no comparable pattern. The consistently used 40-m 6MWT course differs from the standard 30-m course [29, 51]. Dynamic hyperinflation, diaphragm activation, respiratory endurance, daily activity, body composition, inflammatory or metabolic responses, and COPD-specific health status were not measured; mechanistic explanations are therefore physiological interpretations rather than mediation evidence. Strengths include the randomized five-arm comparison, placebo IMT and usual-care controls, standardized four-timepoint assessment, and sensitivity analyses supporting the longitudinal group × time pattern.

## Conclusion

In this single-center randomized trial of women with stable GOLD stage I–II COPD, IMT_AE_ produced the broadest short-term response pattern among the investigated conditions. Improvements were observed across inspiratory muscle strength, inspiratory performance, walking distance, post-exercise dyspnea, and psychological well-being, whereas IMT and AE alone showed more domain-specific responses.

Several outcomes attenuated during the two weeks after supervised training ended, indicating that some early adaptations were only partly retained. These observations describe the early post-intervention course but do not establish the complete time course of detraining or the optimal maintenance strategy.

Overall, IMT_AE_ warrants further evaluation as a short-term training approach in women with stable COPD. Larger, prospectively registered, multicenter randomized trials with longer intervention and follow-up periods are needed to determine the durability and clinical importance of these effects.

## Supplementary Information


Supplementary Material 1.



Supplementary Material 2.



Supplementary Material 3.



Supplementary Material 4.



Supplementary Material 5.



Supplementary Material 6.



Supplementary Material 7.


## Data Availability

Anonymized study results are publicly available in the Figshare repository at https://doi.org/10.6084/m9.figshare.32599071.

## Abbreviations

AE: Aerobic exercise
CONT: Usual-care control group
COPD: Chronic obstructive pulmonary disease
DT1: Seven-day detraining assessment
DT2: Fourteen-day detraining assessment
FEV₁: Forced expiratory volume in one second
FVC: Forced vital capacity
IMT: Inspiratory muscle training
IMT_AE_: Combined inspiratory muscle training and aerobic exercise
IMT_PL_: Placebo inspiratory muscle training
IV: Inspiratory volume
MEP: Maximal expiratory pressure
MIP: Maximal inspiratory pressure
PIFR: Peak inspiratory flow rate
WHO-5: World Health Organization-Five Well-Being Index
6MWT: Six-minute walk test
