# Supplementary material for: Short-term inspiratory muscle training, aerobic exercise, and detraining in women with COPD: a randomized controlled trial
Source: BMC Sports Sci Med Rehabil. 2026 Aug 8;18:390. doi: 10.1186/s13102-026-01964-7 (PMC13536560; doi:10.1186/s13102-026-01964-7)
Supplement: Supplementary file 2 [file 13102_2026_1964_MOESM1_ESM.docx]

**Supplementary Materials**

**Table S1.** Bonferroni-adjusted within-group pairwise comparisons

| **Variable** | **Group** | **Comparison** | **Mean difference** | **Cohen’s dz** | **p Bonf** |
| --- | --- | --- | --- | --- | --- |
| **MIP**  **(cmH₂O)** | **IMT** | Pre–Post | 5.09 | 2.06 | 0.001 |
|  | **IMT** | Pre–DT1 | 3.55 | 1.22 | 0.014 |
|  | **IMT** | Post–DT1 | -1.55 | -1.27 | 0.011 |
|  | **IMT** | Post–DT2 | -3.00 | -1.73 | 0.001 |
|  | **IMT** | DT1–DT2 | -1.45 | -1.06 | 0.033 |
|  | **AE** | Pre–Post | 2.50 | 1.21 | 0.009 |
|  | **AE** | Pre–DT1 | 2.00 | 1.25 | 0.007 |
|  | **AE** | Pre–DT2 | 1.25 | 0.97 | 0.038 |
|  | **AE** | Post–DT1 | -0.50 | -0.96 | 0.041 |
|  | **AE** | Post–DT2 | -1.25 | -1.44 | 0.002 |
|  | **AE** | DT1–DT2 | -0.75 | -1.66 | 0.001 |
|  | **IMT_AE_** | Pre–Post | 9.45 | 2.66 | 0.001 |
|  | **IMT_AE_** | Pre–DT1 | 6.91 | 2.43 | 0.001 |
|  | **IMT_AE_** | Pre–DT2 | 6.00 | 2.41 | 0.001 |
|  | **IMT_AE_** | Post–DT1 | -2.55 | -2.72 | 0.001 |
|  | **IMT_AE_** | Post–DT2 | -3.45 | -2.29 | 0.001 |
|  | **IMT_PL_** | Pre–Post | 2.31 | 1.61 | 0.001 |
|  | **IMT_PL_** | Pre–DT1 | 1.62 | 1.55 | 0.001 |
|  | **IMT_PL_** | Pre–DT2 | 1.31 | 1.53 | 0.001 |
|  | **IMT_PL_** | Post–DT1 | -0.69 | -1.44 | 0.001 |
|  | **IMT_PL_** | Post–DT2 | -1.00 | -1.41 | 0.002 |
| **MEP**  **(cmH₂O)** | **IMT** | Pre–Post | 1.82 | 1.07 | 0.038 |
|  | **IMT** | Post–DT1 | -1.00 | -1.11 | 0.031 |
|  | **IMT** | Post–DT2 | -1.45 | -1.51 | 0.006 |
|  | **AE** | Pre–Post | 0.92 | 1.02 | 0.029 |
|  | **AE** | Pre–DT1 | 0.58 | 1.16 | 0.015 |
|  | **AE** | Post–DT2 | -0.58 | -1.16 | 0.015 |
|  | **IMT_AE_** | Pre–Post | 3.18 | 1.14 | 0.027 |
|  | **IMT_AE_** | Pre–DT1 | 2.27 | 1.47 | 0.007 |
|  | **IMT_AE_** | Post–DT2 | -2.09 | -1.69 | 0.003 |
|  | **IMT_AE_** | DT1–DT2 | -1.18 | -1.69 | 0.003 |
| **PIFR**  **(L/s)** | **IMT** | Pre–Post | 0.33 | 6.40 | 0.001 |
|  | **IMT** | Pre–DT1 | 0.23 | 4.45 | 0.001 |
|  | **IMT** | Pre–DT2 | 0.13 | 2.49 | 0.006 |
|  | **IMT_AE_** | Pre–Post | 0.45 | 8.64 | 0.001 |
|  | **IMT_AE_** | Pre–DT1 | 0.31 | 10.26 | 0.001 |
|  | **IMT_AE_** | Pre–DT2 | 0.21 | 6.94 | 0.001 |
|  | **IMT_AE_** | Post–DT1 | -0.15 | -2.76 | 0.001 |
|  | **IMT_AE_** | Post–DT2 | -0.25 | -4.66 | 0.001 |
| **IV**  **(L)** | **IMT** | Pre–Post | 0.15 | 1.11 | 0.030 |
|  | **IMT** | Post–DT2 | -0.04 | -1.08 | 0.034 |
|  | **IMT** | DT1–DT2 | -0.02 | -1.18 | 0.024 |
|  | **AE** | Pre–Post | 0.29 | 2.07 | 0.001 |
|  | **AE** | Pre–DT1 | 0.20 | 2.13 | 0.001 |
|  | **AE** | Pre–DT2 | 0.14 | 2.24 | 0.001 |
|  | **AE** | Post–DT1 | -0.09 | -2.00 | 0.001 |
|  | **AE** | Post–DT2 | -0.15 | -1.94 | 0.001 |
|  | **AE** | DT1–DT2 | -0.06 | -1.84 | 0.002 |
|  | **IMT_AE_** | Pre–Post | 0.33 | 2.54 | 0.001 |
|  | **IMT_AE_** | Pre–DT1 | 0.22 | 2.54 | 0.001 |
|  | **IMT_AE_** | Pre–DT2 | 0.13 | 2.49 | 0.001 |
|  | **IMT_AE_** | Post–DT1 | -0.11 | -2.63 | 0.001 |
|  | **IMT_AE_** | Post–DT2 | -0.20 | -2.61 | 0.001 |
|  | **IMT_AE_** | DT1–DT2 | -0.09 | -2.54 | 0.001 |
| **6MWT Distance**  **(m)** | **IMT** | Pre–Post | 5.09 | 1.75 | 0.003 |
|  | **IMT** | Pre–DT1 | 3.91 | 1.28 | 0.014 |
|  | **IMT** | Pre–DT2 | 3.27 | 1.27 | 0.014 |
|  | **IMT** | Post–DT2 | -1.82 | -1.32 | 0.012 |
|  | **AE** | Pre–Post | 9.67 | 2.35 | 0.001 |
|  | **AE** | Pre–DT1 | 6.83 | 2.47 | 0.001 |
|  | **AE** | Pre–DT2 | 4.42 | 2.84 | 0.001 |
|  | **AE** | Post–DT1 | -2.83 | -2.11 | 0.001 |
|  | **AE** | Post–DT2 | -5.25 | -2.14 | 0.001 |
|  | **AE** | DT1–DT2 | -2.42 | -2.26 | 0.001 |
|  | **IMT_AE_** | Pre–Post | 16.09 | 3.42 | 0.001 |
|  | **IMT_AE_** | Pre–DT1 | 10.45 | 3.35 | 0.001 |
|  | **IMT_AE_** | Pre–DT2 | 6.27 | 3.77 | 0.001 |
|  | **IMT_AE_** | Post–DT1 | -5.64 | -3.53 | 0.001 |
|  | **IMT_AE_** | Post–DT2 | -9.82 | -3.38 | 0.001 |
|  | **IMT_AE_** | DT1–DT2 | -4.18 | -3.25 | 0.001 |
| **FVC**  **(L)** | **IMT** | Post–DT1 | -0.05 | -5.04 | 0.001 |
|  | **IMT** | Post–DT2 | -0.05 | -4.53 | 0.001 |
|  | **AE** | Pre–Post | 0.08 | 1.22 | 0.015 |
|  | **AE** | Pre–DT1 | 0.06 | 1.25 | 0.011 |
|  | **AE** | Pre–DT2 | 0.04 | 1.17 | 0.014 |
|  | **AE** | Post–DT1 | -0.03 | -1.43 | 0.003 |
|  | **AE** | Post–DT2 | -0.04 | -1.48 | 0.002 |
|  | **AE** | DT1–DT2 | -0.02 | -1.36 | 0.005 |
|  | **IMT_AE_** | Pre–Post | 0.12 | 1.27 | 0.013 |
|  | **IMT_AE_** | Pre–DT1 | 0.09 | 1.27 | 0.013 |
|  | **IMT_AE_** | Pre–DT2 | 0.07 | 1.26 | 0.014 |
|  | **IMT_AE_** | Post–DT1 | -0.03 | -1.43 | 0.008 |
|  | **IMT_AE_** | Post–DT2 | -0.05 | -1.33 | 0.012 |
|  | **IMT_AE_** | DT1–DT2 | -0.03 | -1.23 | 0.017 |
| **FEV₁**  **(L)** | **IMT_AE_** | Pre–Post | 0.12 | 1.48 | 0.002 |
|  | **IMT_AE_** | Pre–DT1 | 0.09 | 1.46 | 0.002 |
|  | **IMT_AE_** | Pre–DT2 | 0.06 | 1.46 | 0.002 |
|  | **IMT_AE_** | Post–DT1 | -0.03 | -1.45 | 0.002 |
|  | **IMT_AE_** | Post–DT2 | -0.05 | -1.44 | 0.002 |
|  | **IMT_AE_** | DT1–DT2 | -0.03 | -1.42 | 0.002 |
| **FEV₁/FVC Ratio** | **IMT_AE_** | Pre–Post | 0.01 | 1.11 | 0.025 |
|  | **IMT_AE_** | Pre–DT1 | 0.01 | 1.10 | 0.026 |
|  | **IMT_AE_** | Post–DT2 | -0.00 | -1.08 | 0.030 |
|  | **IMT_AE_** | DT1–DT2 | -0.00 | -0.99 | 0.049 |
| **Modified BORG Dyspnea Score** | **IMT** | Pre–Post | -0.64 | -1.26 | 0.011 |
|  | **IMT** | Pre–DT1 | -0.55 | -1.04 | 0.036 |
|  | **AE** | Pre–Post | -0.58 | -1.13 | 0.014 |
|  | **AE** | Post–DT2 | 0.58 | 1.13 | 0.014 |
|  | **IMT_AE_** | Pre–Post | -1.09 | -3.62 | 0.001 |
|  | **IMT_AE_** | Pre–DT1 | -0.82 | -2.02 | 0.001 |
|  | **IMT_AE_** | Post–DT2 | 0.82 | 2.02 | 0.001 |
|  | **IMT_AE_** | DT1–DT2 | 0.55 | 1.04 | 0.036 |
| **WHO-5 Well-Being Index score** | **IMT** | Pre–Post | 5.82 | 2.79 | 0.001 |
|  | **IMT** | Pre–DT1 | 4.36 | 2.02 | 0.001 |
|  | **IMT** | Post–DT2 | -4.00 | -2.24 | 0.001 |
|  | **IMT** | DT1–DT2 | -2.55 | -1.26 | 0.011 |
|  | **AE** | Pre–Post | 5.33 | 1.71 | 0.001 |
|  | **AE** | Pre–DT1 | 3.67 | 1.78 | 0.001 |
|  | **AE** | Post–DT2 | -4.00 | -1.66 | 0.001 |
|  | **AE** | DT1–DT2 | -2.33 | -1.13 | 0.014 |
|  | **IMT_AE_** | Pre–Post | 9.45 | 4.68 | 0.001 |
|  | **IMT_AE_** | Pre–DT1 | 6.18 | 2.96 | 0.001 |
|  | **IMT_AE_** | Pre–DT2 | 3.27 | 1.36 | 0.007 |
|  | **IMT_AE_** | Post–DT1 | -3.27 | -2.02 | 0.001 |
|  | **IMT_AE_** | Post–DT2 | -6.18 | -2.96 | 0.001 |
|  | **IMT_AE_** | DT1–DT2 | -2.91 | -1.56 | 0.003 |

Only pairwise comparisons related to significant omnibus time or interaction effects are shown. Mean differences were calculated as the later time point minus the earlier time point. Accordingly, positive values indicate an increase from the earlier to the later assessment, whereas negative values indicate a decrease. Pre indicates the baseline measurement; Post indicates the assessment performed after the four-week intervention; DT1 and DT2 indicate the first and second detraining assessments, respectively. IMT: inspiratory muscle training; AE: aerobic exercise; IMT_AE_: combined inspiratory muscle training and aerobic exercise; CONT: control group; IMT_PL_: placebo inspiratory muscle training group. Adjusted p values were obtained using Bonferroni correction. Within-group effect sizes are reported as Cohen’s dz. A p value <0.05 was considered statistically significant.
