# Supplementary material for: Short-term inspiratory muscle training, aerobic exercise, and detraining in women with COPD: a randomized controlled trial
Source: BMC Sports Sci Med Rehabil. 2026 Aug 8;18:390. doi: 10.1186/s13102-026-01964-7 (PMC13536560; doi:10.1186/s13102-026-01964-7)
Supplement: Supplementary file 3 [file 13102_2026_1964_MOESM2_ESM.docx]

**Supplementary Materials**

**Table S2.** Bonferroni-adjusted between-group pairwise comparisons

| **Variable** | **Time** | **Comparison** | **Mean difference** | **Cohen’s d** | **p Bonf** |
| --- | --- | --- | --- | --- | --- |
| PIFR  (L/s) | Post | IMT–AE | 0.30 | 1.14 | 0.123 |
|  | Post | IMT–IMT_AE_ | -0.07 | -0.31 | 1.000 |
|  | Post | IMT–CONT | 0.42 | 1.55 | 0.013 |
|  | Post | IMT–IMT_PL_ | 0.38 | 1.54 | 0.011 |
|  | Post | AE– IMT_AE_ | -0.37 | -1.41 | 0.028 |
|  | Post | AE–CONT | 0.12 | 0.42 | 1.000 |
|  | Post | AE– IMT_PL_ | 0.08 | 0.28 | 1.000 |
|  | Post | IMT_AE_–CONT | 0.50 | 1.80 | 0.003 |
|  | Post | IMT_AE_– IMT_PL_ | 0.45 | 1.83 | 0.002 |
|  | Post | CONT– IMT_PL_ | -0.05 | -0.17 | 1.000 |
|  | DT1 | IMT–AE | 0.22 | 0.85 | 0.541 |
|  | DT1 | IMT– IMT_AE_ | -0.02 | -0.08 | 1.000 |
|  | DT1 | IMT–CONT | 0.32 | 1.18 | 0.099 |
|  | DT1 | IMT– IMT_PL_ | 0.29 | 1.22 | 0.069 |
|  | DT1 | AE– IMT_AE_ | -0.24 | -0.95 | 0.339 |
|  | DT1 | AE–CONT | 0.10 | 0.33 | 1.000 |
|  | DT1 | AE– IMT_PL_ | 0.07 | 0.25 | 1.000 |
|  | DT1 | IMT_AE_ –CONT | 0.34 | 1.28 | 0.058 |
|  | DT1 | IMT_AE_ – IMT_PL_ | 0.31 | 1.34 | 0.035 |
|  | DT1 | CONT– IMT_PL_ | -0.03 | -0.12 | 1.000 |
|  | DT2 | IMT–AE | 0.20 | 0.76 | 0.824 |
|  | DT2 | IMT– IMT_AE_ | -0.02 | -0.08 | 1.000 |
|  | DT2 | IMT–CONT | 0.27 | 1.03 | 0.228 |
|  | DT2 | IMT– IMT_PL_ | 0.21 | 0.79 | 0.656 |
|  | DT2 | AE– IMT_AE_ | -0.22 | -0.85 | 0.533 |
|  | DT2 | AE–CONT | 0.07 | 0.23 | 1.000 |
|  | DT2 | AE– IMT_PL_ | 0.01 | 0.02 | 1.000 |
|  | DT2 | IMT_AE_–CONT | 0.28 | 1.13 | 0.133 |
|  | DT2 | IMT_AE_– IMT_PL_ | 0.22 | 0.89 | 0.413 |
|  | DT2 | CONT– IMT_PL_ | -0.06 | -0.21 | 1.000 |
| IV  (L) | Post | IMT–AE | -0.19 | -0.84 | 0.582 |
|  | Post | IMT– IMT_AE_ | -0.17 | -0.78 | 0.827 |
|  | Post | IMT–CONT | 0.26 | 1.17 | 0.105 |
|  | Post | IMT– IMT_PL_ | 0.23 | 1.09 | 0.145 |
|  | Post | AE– IMT_AE_ | 0.02 | 0.08 | 1.000 |
|  | Post | AE–CONT | 0.44 | 1.89 | 0.001 |
|  | Post | AE– IMT_PL_ | 0.42 | 1.83 | 0.001 |
|  | Post | IMT_AE_–CONT | 0.42 | 1.86 | 0.002 |
|  | Post | IMT_AE_– IMT_PL_ | 0.40 | 1.80 | 0.002 |
|  | Post | CONT– IMT_PL_ | -0.02 | -0.10 | 1.000 |
|  | DT1 | IMT–AE | -0.12 | -0.57 | 1.000 |
|  | DT1 | IMT– IMT_AE_ | -0.08 | -0.37 | 1.000 |
|  | DT1 | IMT–CONT | 0.24 | 1.08 | 0.172 |
|  | DT1 | IMT– IMT_PL_ | 0.21 | 0.98 | 0.257 |
|  | DT1 | AE– IMT_AE_ | 0.04 | 0.21 | 1.000 |
|  | DT1 | AE–CONT | 0.35 | 1.60 | 0.007 |
|  | DT1 | AE– IMT_PL_ | 0.33 | 1.51 | 0.010 |
|  | DT1 | IMT_AE_ –CONT | 0.31 | 1.43 | 0.025 |
|  | DT1 | IMT_AE_– IMT_PL_ | 0.29 | 1.34 | 0.035 |
|  | DT1 | CONT– IMT_PL_ | -0.03 | -0.12 | 1.000 |
|  | DT2 | IMT–AE | -0.07 | -0.36 | 1.000 |
|  | DT2 | IMT– IMT_AE_ | -0.01 | -0.05 | 1.000 |
|  | DT2 | IMT–CONT | 0.22 | 1.02 | 0.236 |
|  | DT2 | IMT– IMT_PL_ | 0.19 | 0.92 | 0.351 |
|  | DT2 | AE– IMT_AE_ | 0.06 | 0.33 | 1.000 |
|  | DT2 | AE–CONT | 0.29 | 1.36 | 0.031 |
|  | DT2 | AE– IMT_PL_ | 0.26 | 1.27 | 0.043 |
|  | DT2 | IMT_AE_ –CONT | 0.23 | 1.10 | 0.158 |
|  | DT2 | IMT_AE_ – IMT_PL_ | 0.20 | 1.00 | 0.236 |
|  | DT2 | CONT– IMT_PL_ | -0.03 | -0.13 | 1.000 |
| Modified BORG dyspnea score | Post | IMT–AE | -0.08 | -0.15 | 1.000 |
|  | Post | IMT– IMT_AE_ | -0.09 | -0.18 | 1.000 |
|  | Post | IMT–CONT | -0.50 | -1.02 | 0.229 |
|  | Post | IMT– IMT_PL_ | -0.62 | -1.09 | 0.147 |
|  | Post | AE– IMT_AE_ | -0.01 | -0.01 | 1.000 |
|  | Post | AE–CONT | -0.42 | -0.69 | 1.000 |
|  | Post | AE– IMT_PL_ | -0.53 | -0.81 | 0.556 |
|  | Post | IMT_AE_–CONT | -0.41 | -0.77 | 0.788 |
|  | Post | IMT_AE_– IMT_PL_ | -0.52 | -0.87 | 0.451 |
|  | Post | CONT– IMT_PL_ | -0.12 | -0.19 | 1.000 |

Only pairwise comparisons following significant between-group effects at individual measurement time points are shown. Mean differences were calculated as the first group minus the second group in each comparison. Positive values indicate higher values in the first group, whereas negative values indicate lower values in the first group. Post indicates the assessment performed after the four-week intervention; DT1 and DT2 indicate the first and second detraining assessments, respectively. IMT: inspiratory muscle training; AE: aerobic exercise; IMT_AE_: combined inspiratory muscle training and aerobic exercise; CONT: control group; IMT_PL_: placebo inspiratory muscle training group. Adjusted p values were obtained using Bonferroni correction. Between-group effect sizes are reported as Cohen’s d. A p value <0.05 was considered statistically significant.
