# Supplementary material for: Short-term inspiratory muscle training, aerobic exercise, and detraining in women with COPD: a randomized controlled trial
Source: BMC Sports Sci Med Rehabil. 2026 Aug 8;18:390. doi: 10.1186/s13102-026-01964-7 (PMC13536560; doi:10.1186/s13102-026-01964-7)
Supplement: Supplementary file 4 [file 13102_2026_1964_MOESM3_ESM.docx]

**Supplementary Materials**

**Table S3.** Baseline characteristics of completers and non-completers

| **Variable** | **Completers (n = 59)** | **Non-completers (n = 9)** | **p value** |
| --- | --- | --- | --- |
| **Age (years)** | 57.58 ± 4.09 | 55.78 ± 4.21 | 0.257 |
| **Height (cm)** | 159.69 ± 5.17 | 160.16 ± 3.99 | 0.759 |
| **Body mass (kg)** | 67.34 ± 6.60 | 65.01 ± 8.76 | 0.463 |
| **BMI (kg/m²)** | 26.40 ± 2.14 | 25.32 ± 2.90 | 0.311 |
| **MIP (cmH₂O)** | 60.25 ± 9.02 | 60.22 ± 9.54 | 0.993 |
| **MEP (cmH₂O)** | 73.47 ± 6.80 | 71.56 ± 6.21 | 0.412 |
| **PIFR (L/s)** | 3.71 ± 0.27 | 3.74 ± 0.24 | 0.697 |
| **Inspiratory volume (L)** | 2.32 ± 0.20 | 2.39 ± 0.13 | 0.219 |
| **6MWT distance (m)** | 439.45 ± 23.23 | 446.70 ± 17.84 | 0.297 |
| **FVC (L)** | 3.32 ± 0.33 | 3.45 ± 0.31 | 0.273 |
| **FEV₁ (L)** | 2.05 ± 0.24 | 2.15 ± 0.23 | 0.248 |
| **FEV₁/FVC ratio** | 0.62 ± 0.02 | 0.62 ± 0.02 | 0.326 |
| **Post-BD FEV₁ (% predicted)** | 88.86 ± 6.23 | 90.72 ± 3.85 | 0.238 |
| **mMRC dyspnea score** | 0.86 ± 0.75 | 0.67 ± 0.71 | 0.455 |
| **GOLD stage I / II, n** | 55 / 4 | 9 / 0 | Descriptive |
| **mMRC 0 / 1 / 2, n** | 21 / 25 / 13 | 4 / 4 / 1 | Descriptive |

Values are presented as mean ± standard deviation unless otherwise stated. Completers were participants included in the per-protocol analysis, whereas non-completers were randomized participants who discontinued follow-up before completing all planned assessments. Continuous variables were compared using Welch’s independent-samples t tests because of the unequal group sizes. Categorical variables are presented descriptively. BMI: body mass index; MIP: maximal inspiratory pressure; MEP: maximal expiratory pressure; PIFR: peak inspiratory flow rate; 6MWT: six-minute walk test; FVC: forced vital capacity; FEV₁: forced expiratory volume in one second; Post-BD: post-bronchodilator; mMRC: modified Medical Research Council dyspnea scale; GOLD: Global Initiative for Chronic Obstructive Lung Disease.
