# Supplementary material for: Short-term inspiratory muscle training, aerobic exercise, and detraining in women with COPD: a randomized controlled trial
Source: BMC Sports Sci Med Rehabil. 2026 Aug 8;18:390. doi: 10.1186/s13102-026-01964-7 (PMC13536560; doi:10.1186/s13102-026-01964-7)
Supplement: Supplementary file 5 [file 13102_2026_1964_MOESM4_ESM.docx]

Supplementary Materials

**Table S4**. Linear mixed-effects sensitivity analysis with Holm-adjusted group × time interaction tests

| **Outcome** | **LRT χ²** | **df** | **Raw p value** | **Holm-adjusted p value** |
| --- | --- | --- | --- | --- |
| **MIP (cmH₂O)** | 165.687 | 12 | <0.001 | <0.001 |
| **MEP (cmH₂O)** | 100.769 | 12 | <0.001 | <0.001 |
| **PIFR (L/s)** | 340.325 | 12 | <0.001 | <0.001 |
| **IV (L)** | 178.888 | 12 | <0.001 | <0.001 |
| **6MWT distance (m)** | 254.002 | 12 | <0.001 | <0.001 |
| **FVC (L)** | 105.816 | 12 | <0.001 | <0.001 |
| **FEV₁ (L)** | 69.839 | 12 | <0.001 | <0.001 |
| **FEV₁/FVC ratio** | 15.207 | 12 | 0.230 | 0.230 |
| **Post-exercise Borg dyspnea score (0–10)** | 94.231 | 12 | <0.001 | <0.001 |
| **WHO-5 well-being score (0–100)** | 135.833 | 12 | <0.001 | <0.001 |

Linear mixed-effects models with a random intercept for participant were fitted separately for each outcome. Group, time, and the group × time interaction were entered as fixed effects. The contribution of the group × time interaction was examined using likelihood-ratio testing by comparing the full model with a reduced model excluding the interaction term. LRT χ² indicates the chi-square statistic from the likelihood-ratio test, and df indicates the degrees of freedom for this test. Raw p value refers to the unadjusted p value obtained from the likelihood-ratio test. Holm-adjusted p value refers to the p value adjusted across outcome-level interaction tests to address the risk of Type I error inflation. Statistical significance was evaluated using the Holm-adjusted p values at an alpha level of 0.05. This analysis was exploratory and was used as a sensitivity analysis to complement the prespecified mixed repeated-measures ANOVA. MIP: maximal inspiratory pressure; MEP: maximal expiratory pressure; PIFR: peak inspiratory flow rate; IV: inspiratory volume; 6MWT: six-minute walk test; FVC: forced vital capacity; FEV₁: forced expiratory volume in one second; WHO-5: World Health Organization-Five Well-Being Index; LRT: likelihood-ratio test.
