# Supplementary material for: Short-term inspiratory muscle training, aerobic exercise, and detraining in women with COPD: a randomized controlled trial
Source: BMC Sports Sci Med Rehabil. 2026 Aug 8;18:390. doi: 10.1186/s13102-026-01964-7 (PMC13536560; doi:10.1186/s13102-026-01964-7)
Supplement: Supplementary file 6 [file 13102_2026_1964_MOESM5_ESM.docx]

**Supplementary Materials**

**Table S5.** Baseline maintenance inhaled pharmacotherapy in the per-protocol sample by study group

| **Maintenance inhaled regimen** | **IMT**  **(n = 11)** | **AE**  **(n = 12)** | **IMT_AE_**  **(n = 11)** | **CONT**  **(n = 12)** | **IMT_PL_**  **(n = 13)** | **Overall**  **(N = 59)** |
| --- | --- | --- | --- | --- | --- | --- |
| **No maintenance inhaled therapy** | 9 (81.8) | 9 (75.0) | 9 (81.8) | 9 (75.0) | 11 (84.6) | 47 (79.7) |
| **LABA monotherapy** | 1 (9.1) | 0 (0.0) | 0 (0.0) | 0 (0.0) | 0 (0.0) | 1 (1.7) |
| **LABA/LAMA dual therapy** | 1 (9.1) | 3 (25.0) | 2 (18.2) | 3 (25.0) | 2 (15.4) | 11 (18.6) |

Values are presented as n (%), with percentages calculated within study-group columns. Maintenance inhaled treatment categories are mutually exclusive and reflect the regimen documented at baseline. No participant was receiving LAMA monotherapy or an ICS-containing maintenance regimen at baseline. IMT: inspiratory muscle training; AE: aerobic exercise; IMT_AE_: combined inspiratory muscle training and aerobic exercise; CONT: control group; IMT_PL_: placebo inspiratory muscle training; LABA: long-acting β₂-agonist; LAMA: long-acting muscarinic antagonist; ICS: inhaled corticosteroid.
