# Supplementary material for: Short-term inspiratory muscle training, aerobic exercise, and detraining in women with COPD: a randomized controlled trial
Source: BMC Sports Sci Med Rehabil. 2026 Aug 8;18:390. doi: 10.1186/s13102-026-01964-7 (PMC13536560; doi:10.1186/s13102-026-01964-7)
Supplement: Supplementary file 7 [file 13102_2026_1964_MOESM6_ESM.docx]

**Supplementary Materials**

**Table S6.** Supervised intervention exposure and session completion in the per-protocol sample

| **Group** | **n** | **Prescribed, n** | **Completed, mean ± SD** | **Range** | **Completion, n/N (%)** | **12/12 completed,**  **n (%)** |
| --- | --- | --- | --- | --- | --- | --- |
| **IMT** | 11 | 12 | 11.45 ± 0.82 | 10–12 | 126/132 (95.5) | 7 (63.6) |
| **AE** | 12 | 12 | 11.83 ± 0.39 | 11–12 | 142/144 (98.6) | 10 (83.3) |
| **IMT_AE_** | 11 | 12 | 11.64 ± 0.67 | 10–12 | 128/132 (97.0) | 8 (72.7) |
| **IMT_PL_** | 13 | 12 | 11.62 ± 0.51 | 11–12 | 151/156 (96.8) | 8 (61.5) |
| **Overall** | 47 | 12 | 11.64 ± 0.61 | 10–12 | 547/564 (97.0) | 33 (70.2) |

Values are presented as mean ± standard deviation, range, or n/N (%) as indicated. Prescribed refers to the number of supervised sessions scheduled per participant over the four-week intervention period. Completed refers to the number of supervised sessions completed per participant. Completion represents the total number of completed sessions divided by the total number of prescribed sessions within each group. 12/12 completed indicates the number and percentage of participants who attended all prescribed sessions. The control group is not included because no supervised intervention sessions were prescribed. All participants included in this table completed at least 10 of the 12 scheduled sessions and met the attendance criterion for inclusion in the per-protocol analysis. IMT: inspiratory muscle training; AE: aerobic exercise; IMT_AE_: combined inspiratory muscle training and aerobic exercise; IMT_PL_: placebo inspiratory muscle training.
